# Layered Copper-Metallated Covalent Organic Frameworks for Huisgen Reactions

**DOI:** 10.1021/acsami.1c18295

**Published:** 2021-11-03

**Authors:** Ignacio Romero-Muñiz, Pablo Albacete, Ana E. Platero-Prats, Félix Zamora

**Affiliations:** † Departamento de Química Inorgánica, Facultad de Ciencias, Universidad Autónoma de Madrid, Madrid 28049, Spain; ‡ Condensed Matter Physics Center (IFIMAC), Universidad Autónoma de Madrid, Campus de Cantoblanco, 28049 Madrid, Spain; § Instituto de Investigación Avanzada en Ciencias Químicas de la UAM, Universidad Autónoma de Madrid, Campus de Cantoblanco, 28049 Madrid, Spain

**Keywords:** covalent organic framework, local defects, pair distribution function analyses, 1,3-dipolar cycloaddition, copper catalysis

## Abstract

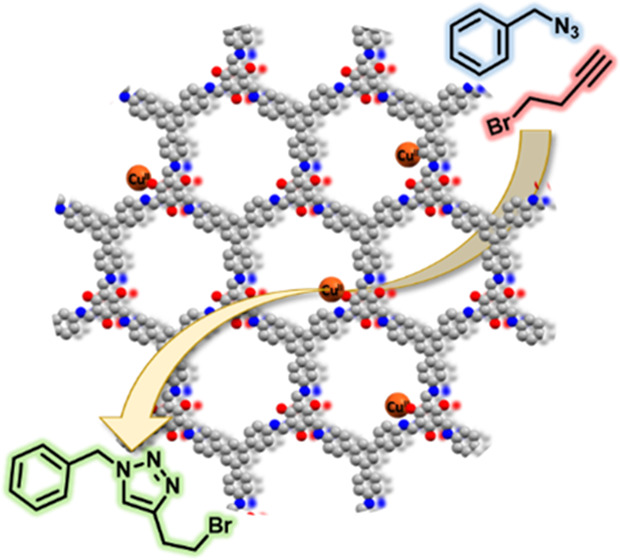

Covalent organic frameworks (COFs) are porous materials formed through condensation reactions of organic molecules *via* the formation of dynamic covalent bonds. Among COFs, those based on imine and β-ketoenamine linkages offer an excellent platform for binding metallic species such as copper to design efficient heterogeneous catalysts. In this work, imine- and β-ketoenamine-based COF materials were modified with catalytic copper sites following a metallation method, which favored the formation of binding amine defects. The obtained copper-metallated COF materials were tested as heterogeneous catalysts for 1,3-dipolar cycloaddition reactions, resulting in high yields and recyclability.

## Introduction

Covalent organic frameworks (COFs) are porous and crystalline materials constructed through reversible condensation reactions between purely organic molecules.^1^ The reticular nature of COF structures and their potential for chemical modifications offer an ideal scenario for catalysis.^2^ In particular, nitrogen-rich COF materials, such as those based on imine-^3,4^ and β-ketoenamine linkages,^5,6^ contain basic binding groups that are able to anchor metals for boosting chemical transformations.^7,8^ In this context, different chemical environments have been widely explored for incorporating metals centers within COF structures: (i) an already metallated building block,^9^ (ii) metal exchange of a previously metallated COF (*i.e.*, porphyrin-based COFs), and (iii) and postsynthetic metallation of a building block.^10^

Copper has been extensively explored as an active site to tune the catalytic properties of materials,^11,12^ in particular COFs.^3^ Especially, attention has been paid to the use of layered COF systems as catalytic platforms, for which both structural flexibility and potential for processing have been demonstrated.^13^ In 2016, Sun et al. developed a β-ketoenamine COF material based on a ditopic linear amine-containing bipyridine group.^14^ The pristine COF itself was catalytically active in the polymerization reactions of phosphonium salts. Interestingly, after the metallation of the bipyridine moieties within the COF with copper(II), the material was found to be catalytically active for the reaction of CO_2_ insertion into epoxides. Copper-containing COF materials have also been proved to catalyze Henry reactions, as shown by Han et al. using a chiral-induced β-ketoenamine framework.^15^ While high conversions were determined for Henry reactions using this chiral COF as a catalyst, significantly low enantiomeric excesses were found. In addition to the aforementioned carbon–carbon coupling, Chan-Lam carbon–nitrogen coupling reactions have also been explored using a copper(II)-loaded polyimide COF as a catalyst. Zhang and co-workers showed the remarkable catalytic performance of this material for a broad scope of aromatic boronic acids and aromatic amines under mild conditions.^16^

The synthesis of 1,2,3 triazole heterocycles, a moiety widely used in coordination and medicinal chemistry,^17,18^ is often achieved through 1,3-dipolar cycloaddition between an alkyne and an azide through the Huisgen reaction. The discovery of the copper(I)-catalyzed variation of this chemical transformation led to an effective and regioselective tool for the synthesis of 1,4-disubstituted 1,3,5-triazoles.^19,20^ This reaction became the paradigm of the “click chemistry”, proceeding under mild conditions with high yields and regioselectivity. Usually, a copper(II) salt is used as a catalytic precursor together with a reducing agent, which *in situ* generates the catalytically active copper(I) species.^21^ However, Kuang et al. reported the possibility of performing this reaction using copper(II) as a catalyst without the need of adding a reducing agent in the reaction mixture.^22^ Under these conditions, the catalytic Cu(I) species are generated by the oxidation of alcoholic media or alkyne homocoupling. Furthermore, Corma and co-workers reported the use of copper(II) metal–organic frameworks to heterogeneously catalyze the Huisgen reaction, with no evidence of the formation of copper(I) species during catalysis. This interesting result pointed toward the possibility of Cu(II) being the catalytically active species for Huisgen reactions within porous frameworks.^23^

Herein, we report the metallation of imine- and β-ketoenamine-based layered COFs with catalytic copper sites. Pair distribution function analyses using synchrotron X-ray total scattering are used to better understand the structural nature of the copper sites within these materials. The obtained COF materials are catalytically active for 1,3-dipolar cycloaddition reactions, showing both high yields and recyclability.

## Results and Discussion

**Figure 1:**
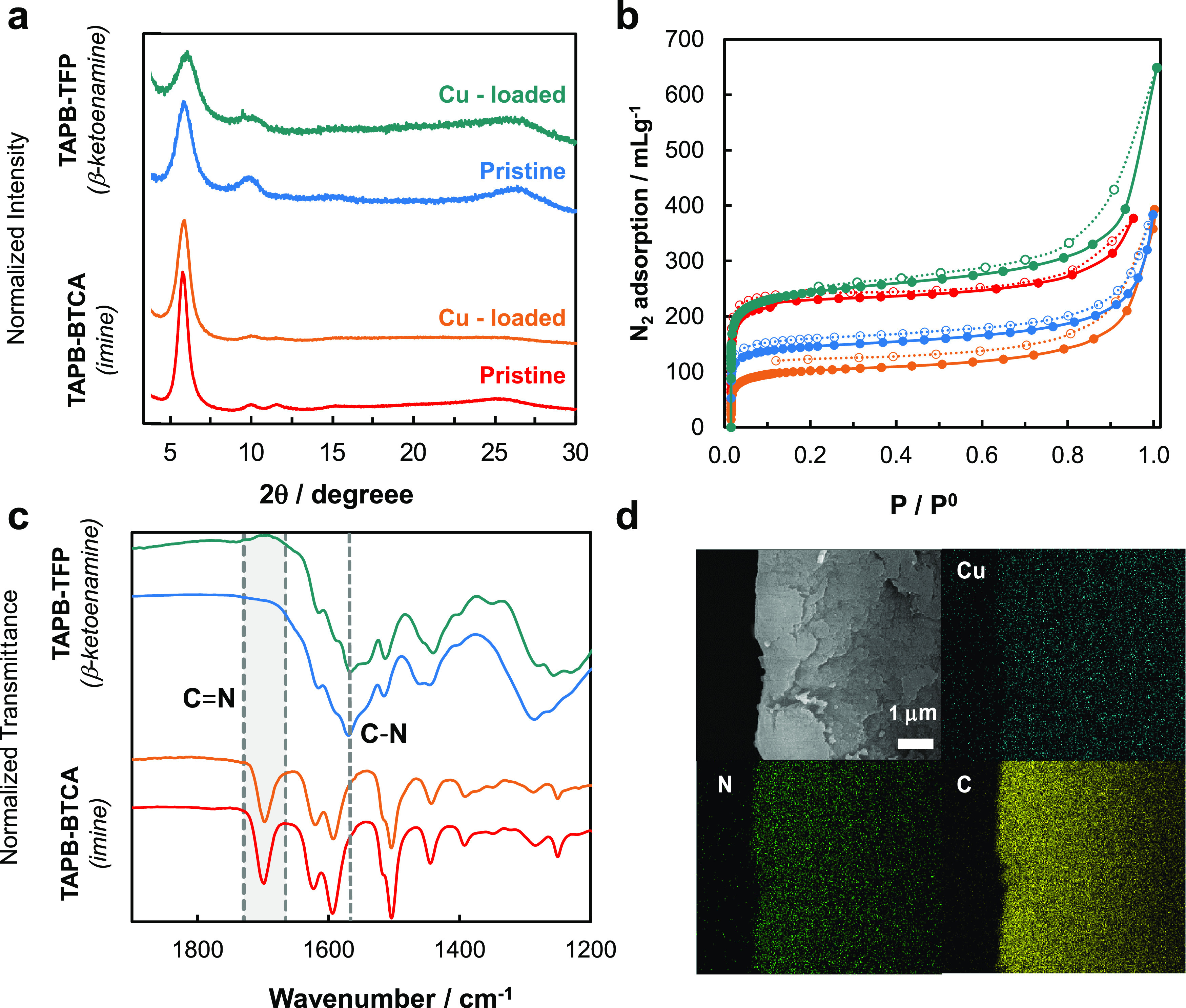
(a) PRXD, (b) N_2_ isotherms at 77 K, and (c) FTIR data collected for pristine and metallated COF materials. (d) SEM-EDX images of Cu-TAPB-TFP.

**Scheme 1:**
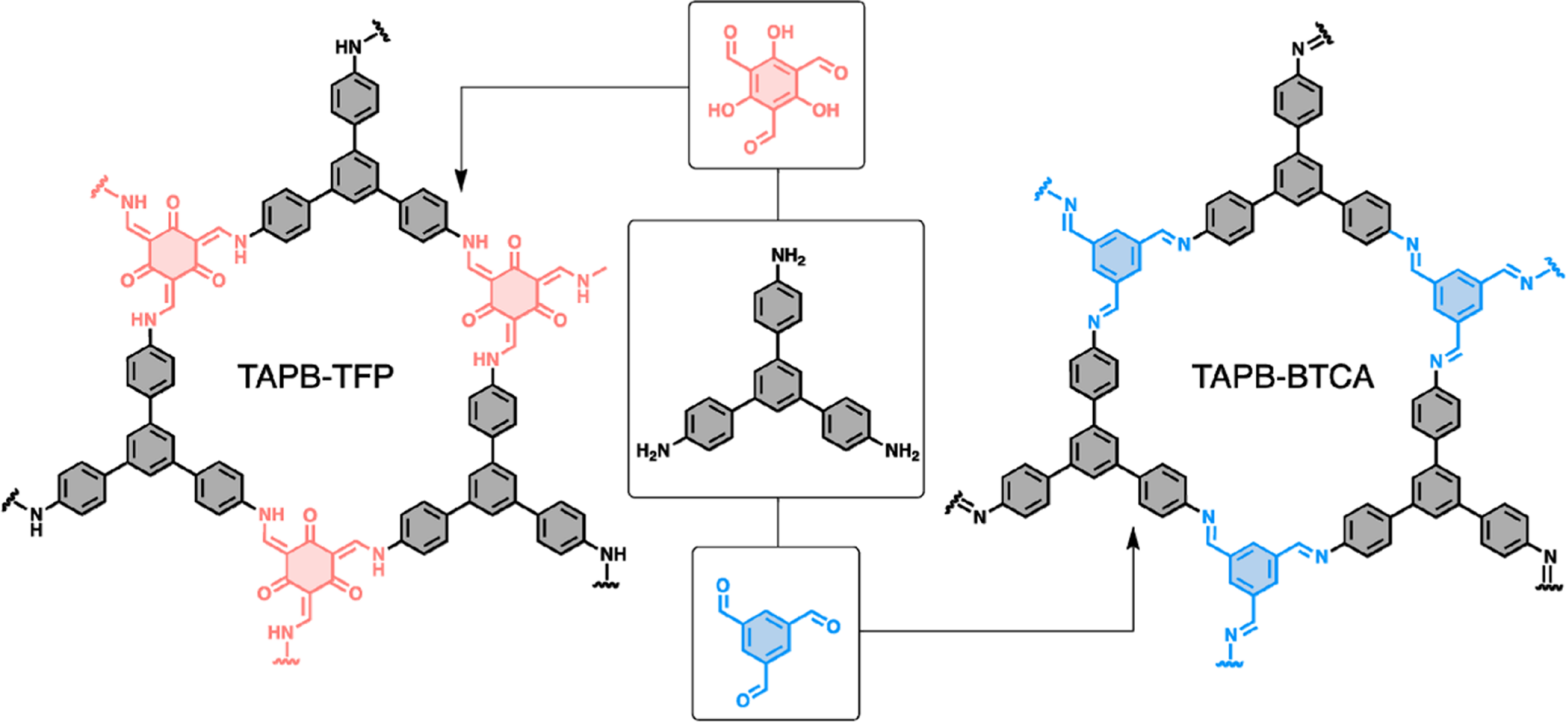
Chemical Representation of the Two-Layered COF Materials Studied in this Work, TAPB-TFP and TAPB-BTCA

The honeycomb layered TAPB-BTCA and TAPB-TFP COFs were prepared by the condensation of 1,3,5-tris-(4′-aminophenyl)benzene (TAPB) with 1,3,5-bencenetricarbaldehyde (BTCA) or 2,4,6-trifromilphloroglucinol (TFP) following the reported procedures^24,25^ (Section S1). The formation of the COF materials was confirmed by solid-state ^13^C cross-polarization magic angle spinning nuclear magnetic resonance (CP-MAS NMR) (Section S2) and attenuated total reflection Fourier transform infrared (ATR-FTIR) spectroscopies (Section S3 and Figure 1). For both materials, the signals linked to aldehyde and amine groups disappeared due to the effective reaction between building blocks. For the TAPB-TFP system, the signals associated with β-ketoenamine bonds were identified, suggesting the majority presence of this tautomer (Figure 1). The crystallinity, or long-range order of the materials, was confirmed by powder X-ray diffraction (PXRD) data with main Bragg peaks at *ca*. 5.8, 10.1, 11.6, and 15.0 2θ degrees corresponding to the (1 0 0), (2 1̅ 0), (2 0 0), and (3 1̅ 0) reflections, respectively, characteristic of both structures (Scheme 1). The porosity of both COF materials was evaluated by measuring nitrogen isotherms at 77 K on the activated systems. Both TAPB-BTCA and TAPB-TFP showed high N_2_ adsorption capacity with Brunauer–Emmet–Teller (BET) surface area values of 888 and 550 m^2^g^–1^, respectively (Section S5).

Under optimized synthesis conditions at room temperature, both COF materials were obtained as gels. We hypothesize that the gel nature of these systems might enhance the diffusion of metal precursors through the COF pores,^26^ resulting in a more homogeneous metallation than when xerogels were used. With this purpose, two different strategies were studied to incorporate copper into the COF structure, as previously explored by us for palladium:^24^ (i) early metallation, for which the copper(II) precursor is added in the early stages of the COF crystallization when the number of defects is significant, and (ii) late metallation, where the copper(II) precursor is added to the COF gel after the crystallization process is complete. The copper content on the metallated COF materials was determined by total reflection X-ray fluorescence (TXRF). Higher copper loadings were systematically found when copper(II) acetate was used compared to other precursors due to its high solubility in the reaction media (Section S4). Regarding the two methods, the early metallation afforded higher copper loadings compared to the late metallation, suggesting the key role of defects to stabilize copper within both the COF systems. Moreover, our results demonstrated that the β-ketoenamine-linked TAPB-TFP COF (0.2 wt %) was able to incorporate higher copper loading than the imine-linked TAPB-BTCA (2.6 wt %). Scanning electron microscopy coupled with energy-dispersive X-ray (SEM-EDX) microanalysis was carried out on the COF samples metallated with copper using the early metallation method (named Cu-TAPB-BTCA and Cu-TAPB-BTCA hereafter) (Figure 1). These analyses demonstrated the homogeneous distribution of copper within the COF structures and the lack of formation of unwanted copper nanoparticles as a byproduct.

The robustness of these layered COF materials upon copper metallation was assessed by PXRD analyses (Figure 1). Our results showed different responses toward metallation depending on the chemical nature of the COF linkages. The imine-linked TAPB-BTCA retained its crystallinity after metallation together with a remarkably low copper loading (*i.e*., 0.2 wt %). On the contrary, the β-ketoenamine-linked TAPB-TFP system could uptake a large amount of copper (*i.e*., 2.6 wt %). These results suggested that copper had more affinity than imine groups to bind amine, in agreement with their higher basic character. For TAPB-BTCA, we suggested that copper was exclusively bonded to the amine defects formed during the COF crystallization, resulting in significantly low copper loadings.

Nitrogen isotherms of the COF materials after copper metallation showed different porosity depending on the chemistry of the pristine systems. On one hand, the imine-linked Cu-TAPB-BTCA showed an expected decrease in the BET surface area to 393 m^2^ g^–1^ compared to 888 m^2^ g^–1^ of the pristine material. TAPB-BTCA showed the main pore size of 12.0 Å (Section S5), with two less populated contributions at 15.6 Å and 19.5 Å. After metallation, the population of the main pore decreased significantly, in agreement with the copper centers pointing toward the pores. On the contrary, Cu-TAPB-TFP showed increased surface area after metallation, from 550 m^2^ g^–1^ for the pristine material to 920 m^2^ g^–1^ for the metallated one. The pristine TAPB-TFP showed the main pore size of 11.7 Å together with two less populated contributions at 15.0 and 19.2 Å. Remarkably, after metallation, a well-defined main pore size at 10.5 Å was observed. While retaining the average structure as demonstrated by PXRD studies, we suggest that the binding of copper sites to the enamine groups within the TAPB-TFP would cause a subtle structural rearrangement of the COF, thereby enhancing the porosity of the material.

**Figure 2:**
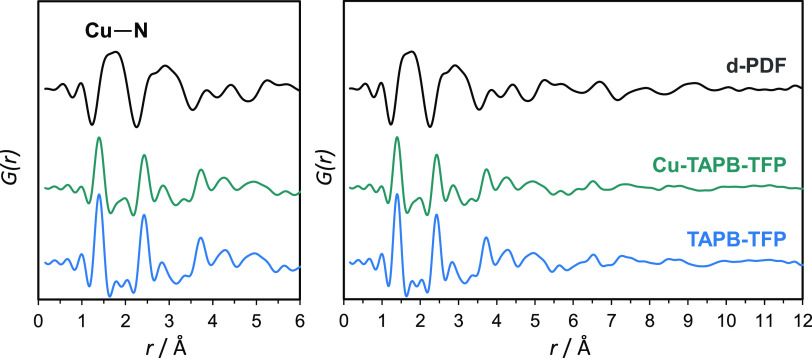
Total PDF data obtained for Cu-TAPB-TFP (green) and TAPB-TFP (blue) materials. d-PDF signal (black) of Cu-TAPB-TFP is obtained after subtraction of the total PDF data of the copper-metallated system to that of the pristine COF.

To gain structural information about the coordination environment of the copper sites within these COF systems, synchrotron X-ray pair distribution function (PDF) studies were performed. The X-ray PDF technique provided precise structural information of porous materials at the local scale,^27−30^ making it a useful tool to characterize the structure of metallated COFs. By subtracting the total PDF data of a pristine COF to that of its metallated counterpart, the new atom–atom distances formed during postsynthetic modification were highlighted. For Cu-TAPB-BTCA, the amount of copper was not high enough to identify the new copper signals by PDF analyses. Differential PDF (d-PDF) analyses performed on Cu-TAPB-TFP (Figure 2) showed a broad contribution at ∼2.0 Å, attributed to Cu–N bonds. The lack of contribution at 2.4 Å, linked to the Cu···Cu distance in the paddle-wheel structure of the copper(II) acetate, demonstrated the absence of unreacted copper salt trapped inside the pores. Small contributions observed at further distances indicated that the copper species were distributed within the COF structure following certain ordering.

**Figure 3:**
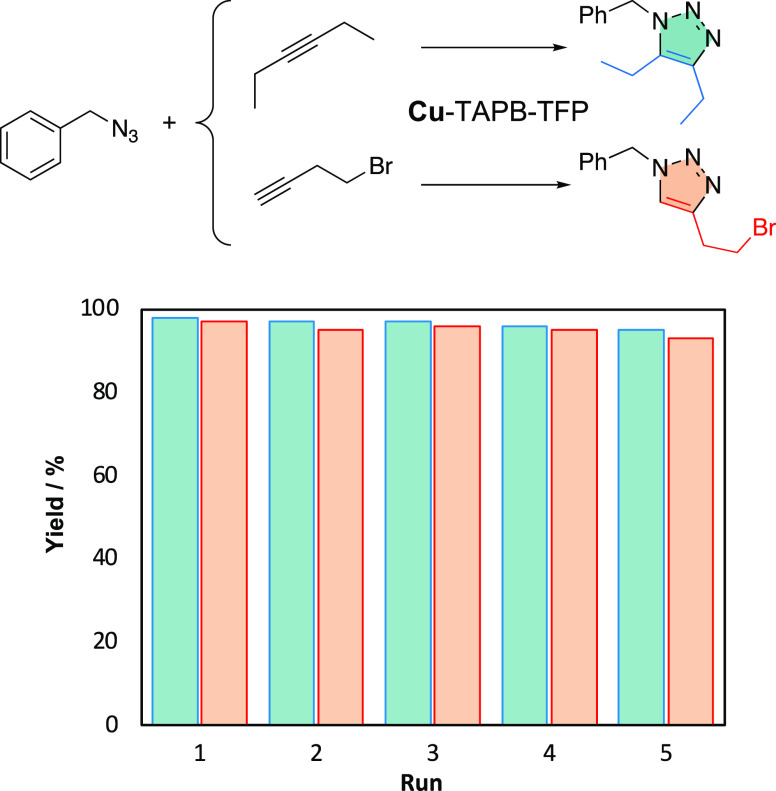
Recycling test of Cu-TAPB-TFP for asymmetric and symmetric cycloaddition reactions.

To evaluate the accessibility of the added copper in Cu-TAPB-TFP *via* the early metallation method, the catalytic activity of this material was tested for the Huisgen [2 + 3] dipolar cycloaddition of alkynes and azides for the formation of 1,2,3 triazoles. As a proof of concept, the reaction of benzyl azide with two different alkynes (*i.e*., the symmetric 3-hexyne and the asymmetric 4-bromobutyne) was evaluated (Table 1). The latter alkyne was chosen to evaluate the regioselectivity of the reaction. Both products were obtained in high yields, over 90%. No reaction product was found when the pristine material was used as a blank. In addition to the high yields obtained, Cu-TAPB-TFP catalyzed the reaction between the asymmetric 4-bromobutyne and the benzyl azide with a 100% regioselectivity to 1,4-isomer. Similar results were obtained when Cu-TAPB-BTCA was used (Table 1). Remarkably, Cu-TAPB-TFP exhibited a high turnover number (TON) of 978. Hot filtration experiments demonstrated the absence of leached copper species during the reaction (Section S7). Furthermore, the recyclability of these COF materials was evaluated by reusing the same catalyst up to five times. Remarkably, no significant decrease in the selectivity nor catalytic activity was observed after five catalytic cycles (Figure 3 and Section S6). The excellent catalytic performance seen together with the robustness of this material suggests strong bonding between enamine groups and the catalytic copper(II) sites.

**Table 1:**
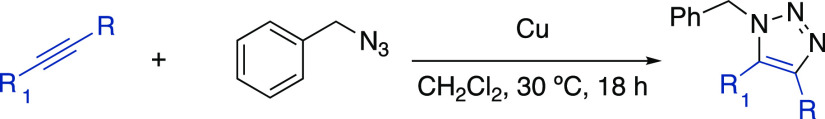

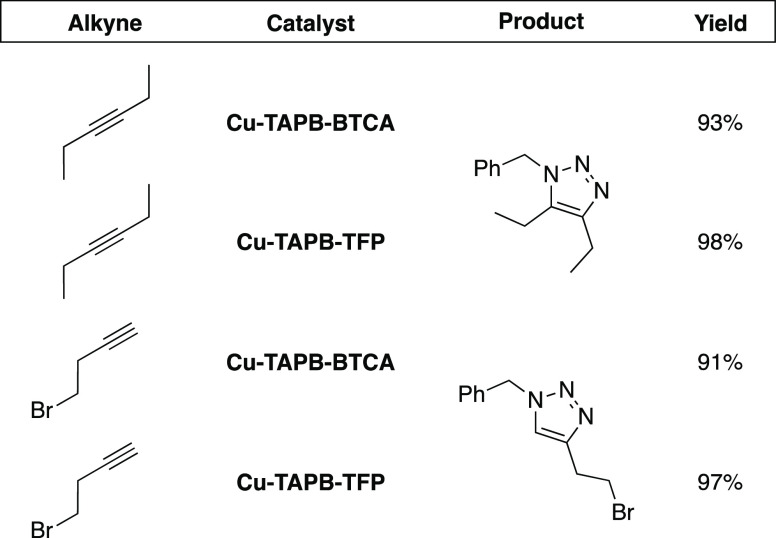
Substrate Scope for the [2 + 3] Dipolar Cycloaddition of Alkynes and Azides using Cu-COFs as the Catalyst

## Conclusions

In this work, two-layered COF materials based on imine- and β-ketoenamine linkages, named TAPB-BTCA and TAPB-TFP, respectively, were metallated with copper using different methods. For both materials, our results confirmed that the incorporation of copper(II) into these nitrogen-rich COF structures was enhanced when performing the metallation on the defective systems (*i.e*., early metallation) compared to the metallation of crystalline COFs as gels. Interestingly, the TAPB-TFP system showed a higher capacity to load copper than TAPB-BTCA, suggesting enamine groups to be optimal binding sites to stabilize cooper(II) sites within COF structures. The catalytic properties of the prepared copper-metallated COFs were evaluated for the 1,3-dipolar cycloaddition reaction between azides and alkynes. Under mild conditions, both materials exhibited 100% regioselectivity to the 1,4-disubstituted product and high catalytic activity with excellent yields. The robustness of the catalytic systems was assessed by performing leaching studies, which showed no copper loss in solution during the reaction. Furthermore, the prepared COF catalysts were recycled up to five times without any significant loss of catalytic activity. We believe that this work opens new opportunities for the development of defective COF materials as supports for active metal sites for heterogeneous catalysis.

## Experimental Section

### *Copper Metallation of TAPB-TFP*

#### Early Metallation

A solution of 40 mg of 1,3,5-tris(4′-aminophenyl)benzene (0.114 mmol) in 2 mL of *m*-cresol and 0.5 mL of glacial acetic acid was added to a solution of 24 mg of 2,4,6-trihydroxibenzene-1,3,5-tricarbaldehyde (0.114 mmol) in 3 mL of *m*-cresol to yield a dark yellow gel. The reaction mixture was undisturbed for 1 h at 30 °C. Then, a solution of the corresponding copper(II) salt (Table S1) was added to the gel. The reaction was kept for 72 h at 30 °C. The resulting green gel was washed with water, tetrahydrofuran, and methanol, filtrated, and air-dried (48 h) to yield the product.

#### Late Metallation

A solution of 40 mg of 1,3,5-tris(4′-aminophenyl)benzene (0.114 mmol) in 2 mL of *m*-cresol and 0.5 mL of glacial acetic acid was added to a solution of 24 mg of 2,4,6-trihydroxibenzene-1,3,5-tricarbaldehyde (0.114 mmol) in 3 mL of *m*-cresol to yield a dark yellow gel. The reaction mixture was undisturbed for 1 h at 30 °C. Then, a solution of the corresponding copper(II) salt (Table S1) was added to the gel. The reaction was maintained for 48 h at 30 °C. The resulting green gel was washed with water, tetrahydrofuran, and methanol, filtrated, and air-dried (48 h) to yield the product.

### *Attenuated Total Reflectance Fourier Transform Infrared Spectroscopy (ATR-FT-IR)*

ATR-FT-IR spectra were recorded using a Perkin Elmer Spectrum 100 with a PIKE Technologies MIRacle Single Reflection Horizontal ATR Accessory with a spectral range of 4000–500 cm^–1^.

#### Solid-State ^13^C CP-MAS Solid-State Nuclear Magnetic Resonance Spectroscopy

Solid-State ^13^C CP-MAS nuclear magnetic resonance spectroscopy was carried out using a Bruker AV 400 WB spectrometer. The ^13^C chemical shifts were given relative to tetramethylsilane as zero ppm.

#### Powder X-ray Diffraction (PXRD)

PXRD data were collected using a Panalytical X’Pert PRO diffractometer with Ge primary monochromator and X′Celerator fast detector. Monochromated copper radiation (working wavelength Kα_1_ = 1.5406 Å) was employed. The samples were scanned in the range from 2θ = 2–30° with a step size of 0.0167° and a step time of 100 s.

#### Nitrogen Adsorption–Desorption Isotherms

Nitrogen adsorption–desorption isotherms were measured using a Micromeritics ASAP2020 volumetric instrument under static adsorption conditions. Before the measurement, powdered samples were heated at 323 K overnight and outgassed to 10^–6^ Torr. The Brunauer–Emmet–Teller (BET) and Langmuir analyses were carried out to determine the total specific surface areas for the N_2_ isotherms at 77 K. Using the nonlocal density functional theory (NLDFT) model, the pore volume was derived from the sorption curve.

#### Total X-ray Reflection Fluorescence (TXRF)

Qualitative and quantitative TXRF analyses for copper content determinations were performed with a benchtop S2 PicoFox TXRF spectrometer from Bruker Nano (Germany), equipped with a Mo X-ray source working at 50 kV and 600 μA, a multilayer monochromator with 80% of reflectivity at 17.5 keV (Mo Kα), an XFlash SDD detector with an effective area of 30 mm^2^, and an energy resolution better than 150 eV for 5.9 keV (Mn Kα). The acquisition time for qualitative analysis was 300 s and that for the quantitative analysis was 500 s.

#### Scanning Electron Microscopy with Energy-Dispersive X-ray Microscopy (SEM-EDX)

SEM-EDX images and EDX spectra were taken in a Hitachi S-3000N microscope with an ESED detector coupled to an INCAx-sight EDX analyzer. For this technique, the samples were metallized with a 15 nm thick Au layer at a pressure of 10^–3^ Pa.

#### ^1^H Nuclear Magnetic Resonance (^1^H NMR)

^1^H NMR spectra were recorded in the CDCl_3_ solution on a 300 MHz Bruker Advance II NMR spectrometer. Chemical shifts were reported in parts per million (ppm), reported at the CDCl_3_ solvent peak, defined at δ = 7.26 ppm. ^1^H NMR splitting patterns were designated as singlet (s), doublet (d), triplet (t), quadruple (q), quintet (p), and double doublet (dd). Signals that could not be easily interpreted or visualized were designated as multiplet (m). Coupling constants (J) are indicated in Hertz.

#### Pair Distribution Function (PDF) Analyses

Total X-ray scattering data suitable for PDF analyses were collected at the P02.1 beamline at a Petra-III synchrotron using 60.0 keV (0.2068 Å) X-rays. PDFs were obtained from the data within xPDFsuite^31^ to a *Q*_max_ = 22 Å^–1^. Differential PDFs were calculated by subtracting the PDF of the pristine COF from that of the metallated material after applying a normalization factor.

#### Catalytic Tests

Fifty microliters (0.40 mmol) of benzyl azide was added to a suspension of 0.40 mmol of the alkyne and 5 mg of the corresponding COF in dichloromethane (1.5 mL) in a capped vial. The reaction mixture was stirred at 30 °C for 18 h. Then, the solvent was evaporated in vacuo. ^1^H NMR analyzed the resulting oil.

## Supporting Information Available

Synthetic procedures, attenuated total reflection Fourier transform infrared (ATR-FTIR) spectroscopy, solid-state ^13^C cross-polarization magic angle spinning nuclear magnetic resonance (CP-MAS NMR), total X-ray reflection fluorescence (TXRF), nitrogen adsorption–desorption isotherms, catalytic and leaching studies, and postcatalytic powder X-ray diffraction and field emission–scanning electron microscopy (PDF)
