# Supplementary material for: Layered Copper-Metallated Covalent Organic Frameworks for Huisgen Reactions
Source: ACS Appl Mater Interfaces. 2021 Nov 3;13(45):54106–12. doi: 10.1021/acsami.1c18295 (PMC8659373; doi:10.1021/acsami.1c18295)
Supplement: Supplementary file 1 — am1c18295_si_001.pdf [file am1c18295_si_001.pdf]

Supporting information

Layered Copper-Metallated Covalent Organic Frameworks for Huisgen Reactions

Ignacio Romero-Muñiz,[a] Pablo Albacete,[a] Ana E. Platero-Prats,*[a,b] Félix Zamora,*[a,b,c]

[a] Departamento de Química Inorgánica, Facultad de Ciencias, Universidad Autónoma de Madrid, Madrid 28049, Spain

[b] Condensed Matter Physics Center (IFIMAC), Universidad Autónoma de Madrid, Campus de Cantoblanco, 28049 Madrid, Spain

[c] Instituto de Investigación Avanzada en Ciencias Químicas de la UAM, Universidad Autónoma de Madrid, Campus de Cantoblanco, 28049 Madrid,

Corresponding Author

E-mail: ana.platero@uam.es, felix.zamora@uam.es.

Supporting Information

S1 Synthetic Procedures S-2

S2 Attenuated Total Reflection Fourier-Transform Infrared (ATR-FTIR) Spectroscopy....... S-4

S3 Solid-State ^{13}C Cross-Polarization Magic Angle Spinning Nuclear Magnetic Resonance (CP-MAS NMR)....... S-6

S4 Total X-ray reflection fluorescence S-8

S5 Nitrogen adsorption-desorption isotherms S-9

S6 Catalytic Studies S-10

S7 Leaching Studies....... S-13

S8 Postcatalytic Powder X Ray Diffraction and FE-SEM S-15

References....... S-17

S1 Synthetic Procedures

All other reagents were used as received from commercial suppliers unless otherwise stated. Reaction progress was monitored by **thin-layer chromatography** (TLC) performed on aluminum plates coated with silica gel F_{254} with 0.2 mm thickness. Chromatograms were visualized by fluorescence quenching with UV light at 254 nm or by staining using potassium permanganate.

1,3,5-tris-(4-aminophenyl)benzene; 2,4,6-trihydroxybenzene-1,3,5-tricarbaldehyde, TAPB-BTCA, and TAPB-TFP were prepared by following reported procedures.[1]

Cu-TAPB-BTCA *early* metallation

A solution of 40 mg of 1,3,5-tris(4'-aminophenyl)benzene (0.114 mmol) in 5.7 mL of glacial acetic acid was added to a solution of 18.4 mg of benzene-1,3,5-tricarbaldehyde (0.114 mmol) in 5.7 mL of glacial acetic acid yielding a dark yellow gel. The reaction mixture was kept for 1 h at 30 ºC. Then a solution of the corresponding copper (II) salt (table S2.1) was added to the gel. The reaction was kept for 72 h at 30 ºC. The resulting green gel was washed with water, tetrahydrofuran, and methanol, filtrated, and air-dried (48 h), yielding the product.

Cu-TAPB-BTCA *late* metallation

A solution of 40 mg of 1,3,5-tris(4'-aminophenyl)benzene (0.114 mmol) in 5.7 mL of glacial acetic acid was added to a solution of 18.4 mg of benzene-1,3,5-tricarbaldehyde (0.114 mmol) in 5.7 mL of glacial acetic acid yielding a dark yellow gel. The reaction mixture was kept for 72 h at 30ºC. Then a solution of the corresponding copper (II) salt (table S2.1) was added to the gel. The reaction was kept for 48 h at 30 ºC. The resulting green gel was washed with water, tetrahydrofuran, and methanol, filtrated and air dried (48 h) yielding the product.

Table S1. Cu (II) salts and solvents used, total yield after loadings, and Cu(II) loading.

Precursor	alt amount	Solvent	Resulting material	eld (%)
**Cu-TAPB-BTCA *early* loading**				
**Precursor**	**alt amount**	**Solvent**	**Resulting material**	**eld (%)**
$CuCl_2$	21 mg<br>0.15 mmol)	Methanol<br>(1.45 mL)	CuCl-TAPB-BTCA-is	72
$CuBr_2$	21 mg<br>0.09 mmol)	Methanol<br>(1.45 mL)	CuBr-TAPB-BTCA-is	71
$Cu(AcO)_2$	21 mg<br>0.10 mmol)	cetic Acid<br>(2.0 mL)	CuAc-TAPB-BTCA-is	71
**Cu-TAPB-TFP *early* loading**				
**Precursor**	**alt amount**	**Solvent**	**Resulting material**	**eld (%)**
$CuCl_2$	21 mg<br>0.15 mmol)	Methanol<br>(1.45 mL)	CuCl-TAPB-TFP-is	70
$CuBr_2$	21 mg<br>0.09 mmol)	Methanol<br>(1.45 mL)	CuBr-TAPB-TFP-is	67
$Cu(AcO)_2$	21 mg<br>0.10 mmol)	cetic Acid<br>(2.0 mL)	CuAc-TAPB-TFP-is	68
**Cu-TAPB-BTCA late loading**				
**Precursor**	**alt amount**	**Solvent**	**Resulting material**	**eld (%)**
$CuCl_2$	21 mg<br>0.15 mmol)	Methanol<br>(1.45 mL)	CuCl-TAPB-BTCA-gl	68
$CuBr_2$	21 mg<br>0.09 mmol)	Methanol<br>(1.45 mL)	CuBr-TAPB-BTCA-gl	69
$Cu(AcO)_2$	21 mg<br>0.10 mmol)	cetic Acid<br>(2.0 mL)	CuAc-TAPB-BTCA-gl	71
**Cu-TAPB-TFP late loading**				
**Precursor**	**alt amount**	**Solvent**	**Resulting material**	**eld (%)**
$CuCl_2$	21 mg<br>0.15 mmol)	Methanol<br>(1.45 mL)	CuCl-TAPB-TFP-gl	65
$CuBr_2$	21 mg<br>0.09 mmol)	Methanol<br>(1.45 mL)	CuBr-TAPB-TFP-gl	64
$Cu(AcO)_2$	21 mg<br>0.10 mmol)	cetic Acid<br>(2.0 mL)	CuAc-TAPB-TFP-gl	66

S2 Attenuated Total Reflection Fourier-Transform Infrared (ATR-FTIR) Spectroscopy.

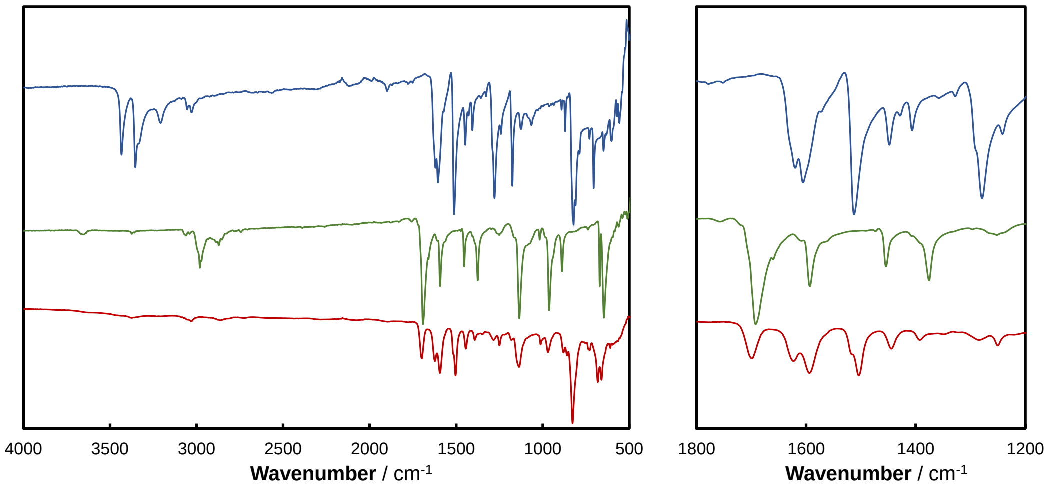

Figure S1. ATR-FT-IR spectra of TAPB (blue), BTCA (green), and **TAPB-BTCA** (red).

In the case of **TAPB-BTCA**, the characteristic ATR-FTIR signals at 1621 and 1279 cm^{-1} corresponding to C=N and –C=N−C stretching bands, respectively, could be observed. Additionally, the complete condensation between the two building blocks can be confirmed by the disappearance of the signal at 1690 cm^{-1} and the signals between 3350 and 3200 cm^{-1} assigned to the aldehyde carbonyl and amine stretching bands, respectively.

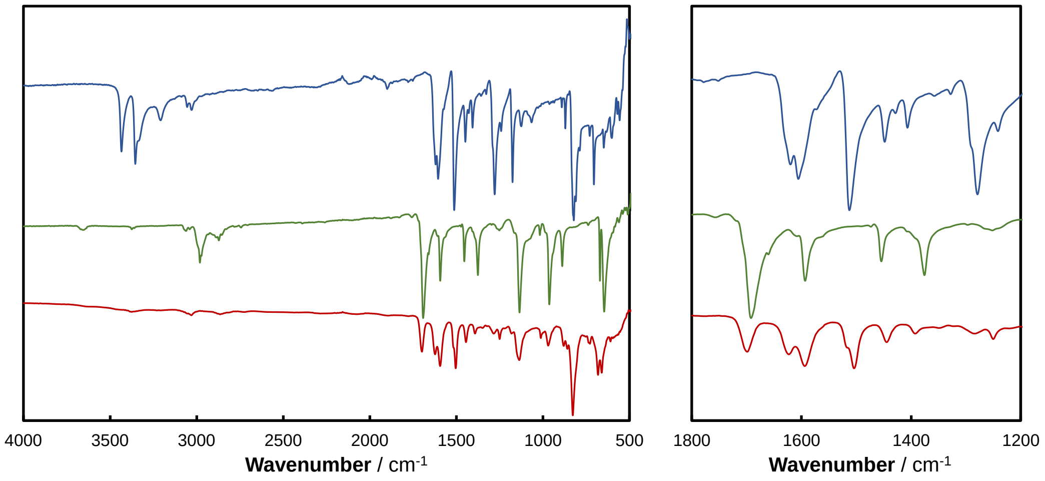

Figure S2. ATR-FT-IR spectra of TAPB (blue), TPP (green), and **TAPB-TFP** (red).

With regard to **TAPB-TFP** ATR-FTIR collected data, two important signals could be found. First, the signal associated with the C=O stretching of the aldehyde shifts from 1658 to 1610 cm^{-1}, corresponding to a C=O stretching signal of the ketone in the backbone of the COF. On the other hand, a signal at 1286 cm^{-1} corresponding to an enamine C–N stretching band appeared.

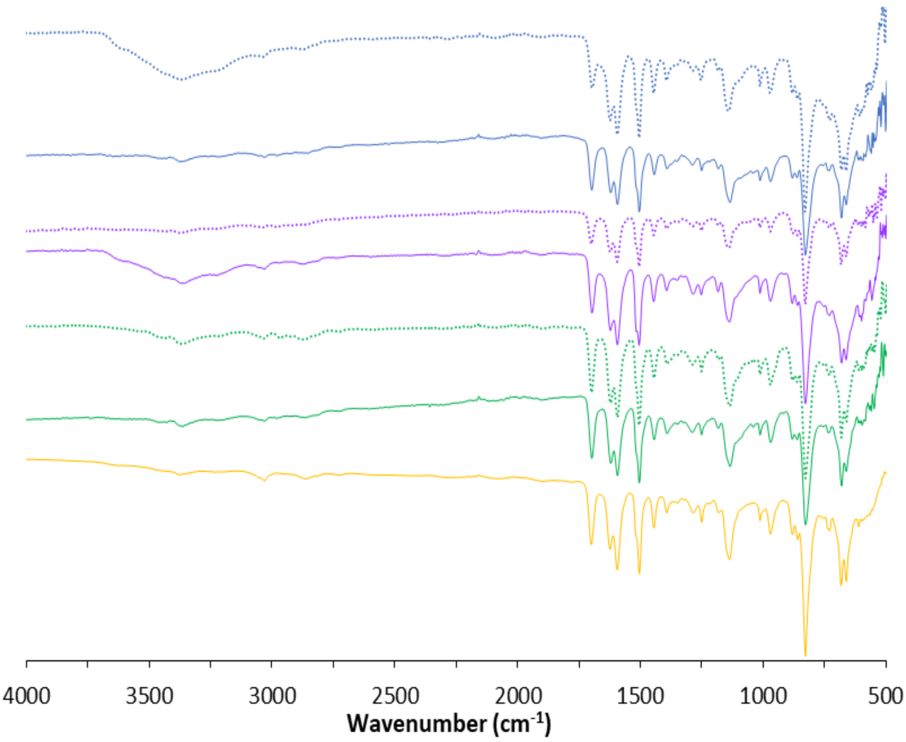

Figure S3. ATR-FT-IR spectra of pristine **TAPB-BTCA** (yellow) metallated with $CuCl_2$ (green), $CuBr_2$ (purple), and $[Cu(CH_3CO_2)_2 \cdot H_2O]_2$ (blue) by both *early* (solid) and *late* (dashed) methods.

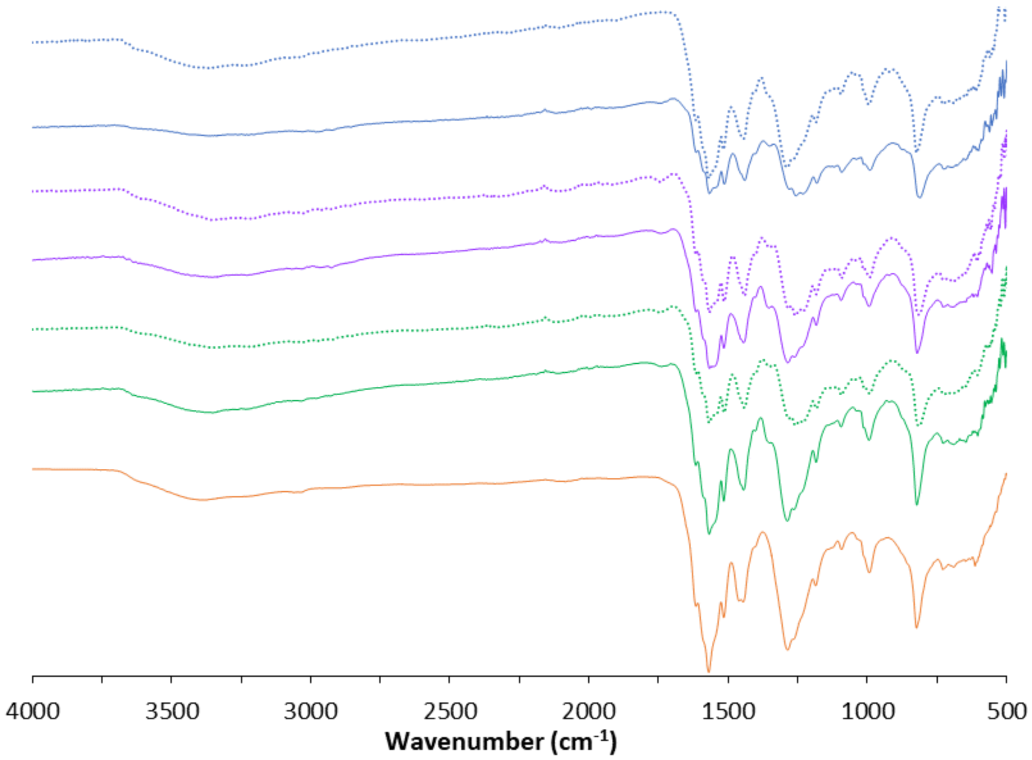

Figure S4. ATR-FT-IR spectra of pristine **TAPB-TFP** (orange) metallated with $CuCl_2$ (green), $CuBr_2$ (purple), and $[Cu(CH_3CO_2)_2{\cdot}H_2O]_2$ (blue) by both *early* (solid) and *late* (dashed) methods.

S3 Solid-State ^{13}C Cross-Polarization Magic Angle Spinning Nuclear Magnetic Resonance (CP-MAS NMR).

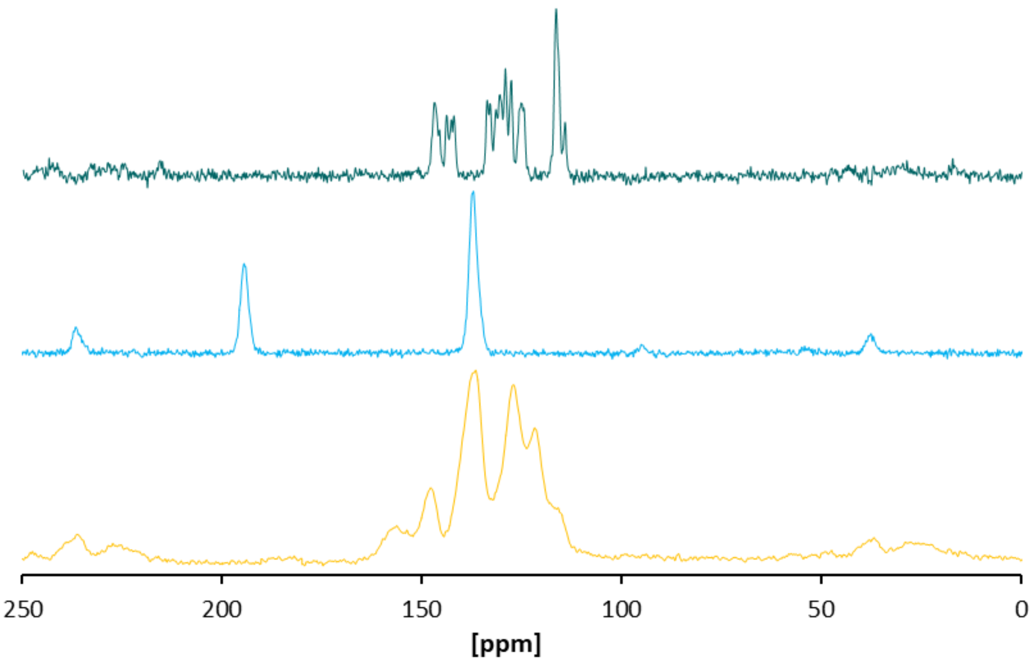

Figure S5. ^{13}C CP-MAS NMR spectra of TAPB (green), BTCA (blue), and **TAPB-BTCA** (yellow).

The ^{13}C CP-MAS NMR data collected on **TAPB-BTCA** showed a resonance at 157 ppm corresponding to an imine carbon atom, while the signal at 192 ppm assigned to benzene-1,3,5-tricabaldehyde carbonyl carbon disappeared, corroborating the condensation of both building blocks.

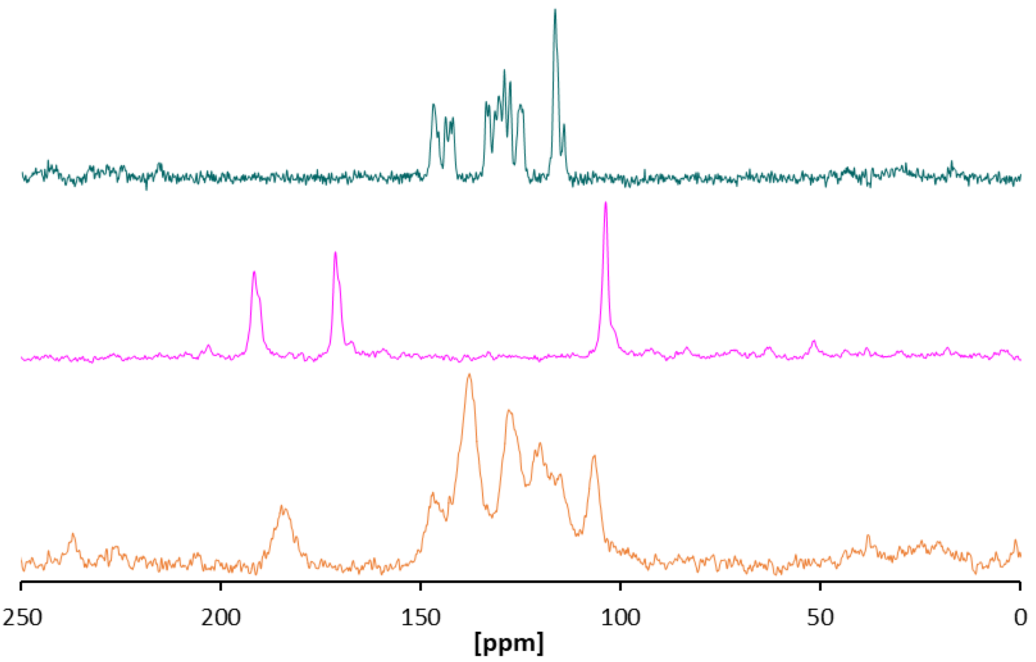

Figure S6. ^{13}C CP-MAS NMR spectra of TAPB (green), TFP (pink), and **TAPB-TFP** (orange).

^{13}C CP-MAS NMR data collected on **TAPB-TFP** showed a signal at 184 ppm assigned to the ketone carbon. Additionally, the signals at 191 and 171 ppm, assigned to the aldehyde and hydroxyl carbon atoms of TFP, respectively, disappeared. The disappearance of the first signal is due to the bonding of the building blocks, while the tautomerization of the hydroxyl group to a ketone causes the disappearance of the latter. In this case, the signal at 147 ppm assigned the aromatic carbon in alpha to the amine remained due, again, to the tautomerization of the imine bond to an amine bond

S4 Total X-ray reflection fluorescence

Table S2. Quantitative TXRF analysis **Cu-RT-COFs**. Weight percentage of Cu (II) loading for the different COFs, salts, and methods used, and nominal loading.

	Precursor	***Early oading***	**Late oading**	***lominal oading***
**PB-BTCA**	$CuCl_2$	0.01	0.01	29.05
	$CuBr_2$	0.03	0.01	29.05
	$Cu(CH_3CO_2)_2{\cdot}H_2O]_2$	0.19	0.04	26.57
**APB-TFP**	$CuCl_2$	2.33	0.40	27.34
	$CuBr_2$	2.10	0.16	27.34
	$Cu(CH_3CO_2)_2{\cdot}H_2O]_2$	2.59	0.94	25.32

S5 Nitrogen adsorption-desorption isotherms

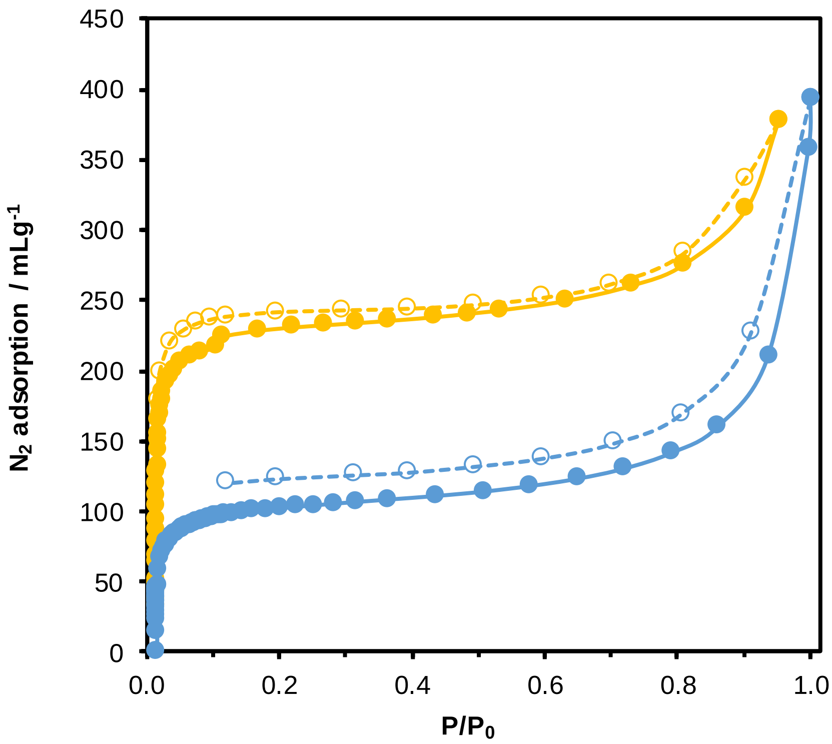

Figure S7. Nitrogen adsorption isotherm of **TAPB-BTCA** (yellow) and **Cu-TAPB-BTCA** (blue).

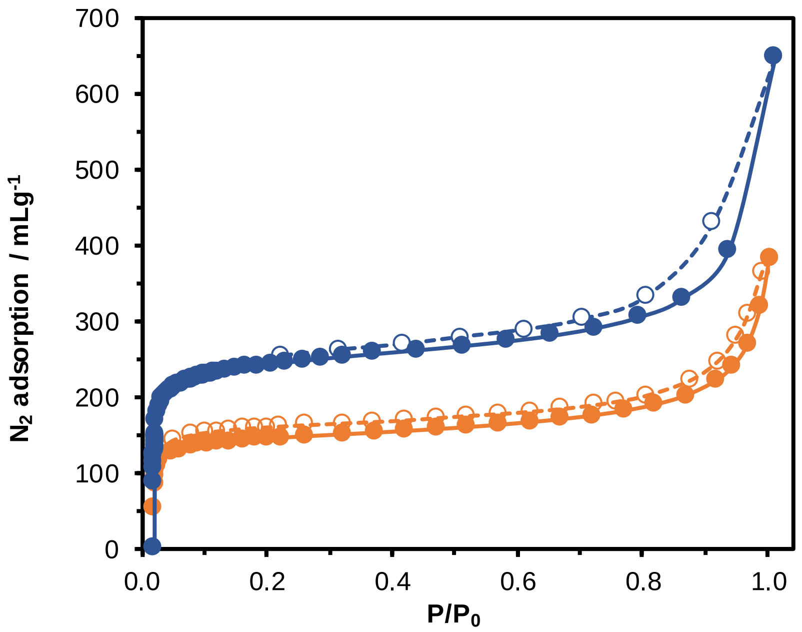

Figure S8. Nitrogen adsorption isotherm of **TAPB-TFP** (orange) and **Cu-TAPB-BTCA** (blue).

S6 Catalytic Studies

58 µL (0.40 mmol) of benzyl azide was added to a suspension of 0.40 mmol of the alkyne and 5 mg of the corresponding COF in dichloromethane (1.5 mL) in a capped vial. The reaction mixture was stirred at 30 ºC for 18 h. Then the solvent was evaporated under vacuum, and the resulting oil was analyzed by 1H NMR. All the signals for the final product are in accordance with the literature [2,3] Same procedure is used for recyclability test adding an additional step of washing the isolated material after the reaction with dichloromethane.

- **1-Phenyl-4-(Bromomethyl)- 1,2,3-triazole**

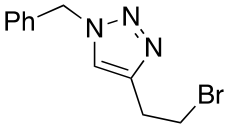

White Solid. ^{1}H NMR (300 MHz, $CDCl_3$): δ 3.25 (t, J = 6.8 Hz, 2H), 3.62 (t, J = 6.8 Hz, 2H), 5.01 (s, 2H), 7.30-7.19 (m, 2H) and 7.32-7.40 (m, 4H).

- **1-benzyl-4,5-(diethyl)-1,2,3-triazole**

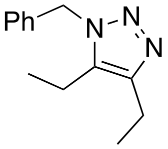

Yellowish oil. ^{1}H NMR (300 MHz, $CDCl_3$) δ 0.96 (t, J = 7.6 Hz, 3H), 1.25 (t, J = 7.6 Hz, 3H), 2.50 (q, J = 7.7 Hz, 2H), 2.60 (q, J = 7.7 Hz, 2H), 5.31 (s, 2H), 7.30-7.46 (m, 5H).

Table S3. Alkyne quantities

Alkyne	nmol	µL	mg
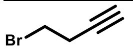	0.40	41.5	[illegible]8.81
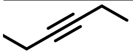	0.40	44.0	31.81

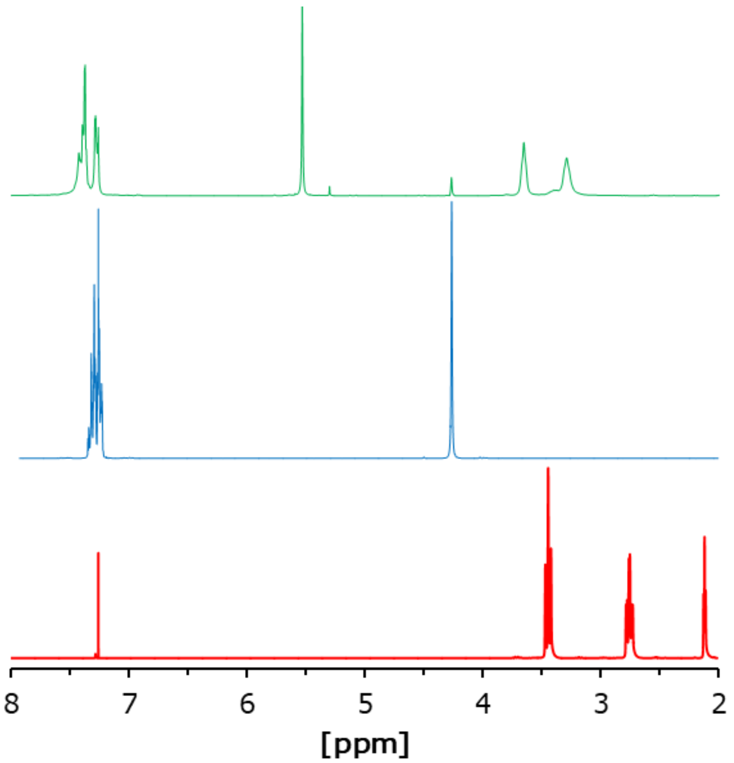

Figure S9. ^{1}H NMR data of [2+3] dipolar cycloaddition with **Cu-TAPB-BTCA** as the catalyst, 4-bromobutyne (red) as alkyne, and benzyl azide (blue). The reaction product (green).

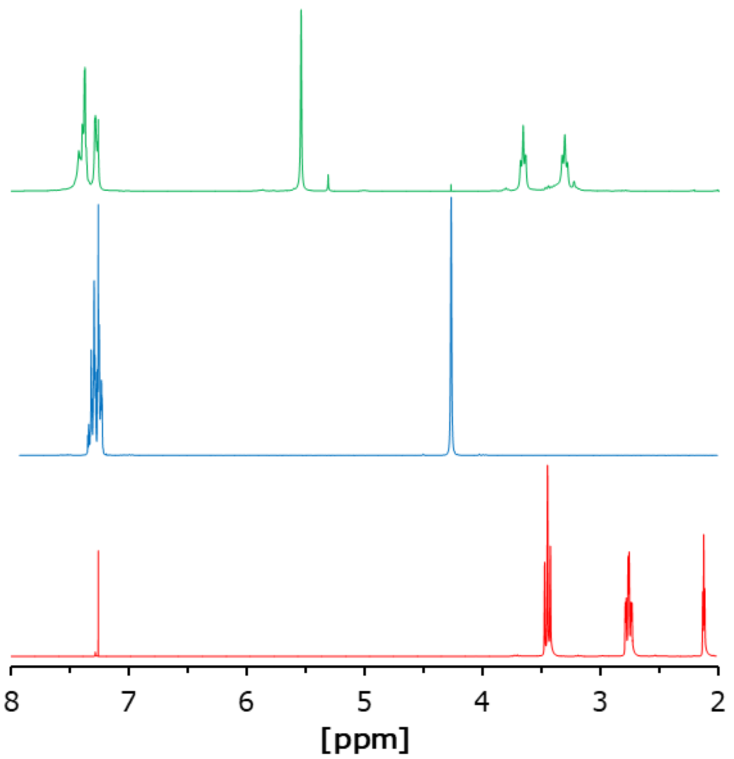

Figure S10. ^{1}H NMR data of [2+3] dipolar cycloaddition with **Cu-TAPB-TFP** as the catalyst, 4-bromobutyne (red) as alkyne, and benzyl azide (blue). The reaction product (green).

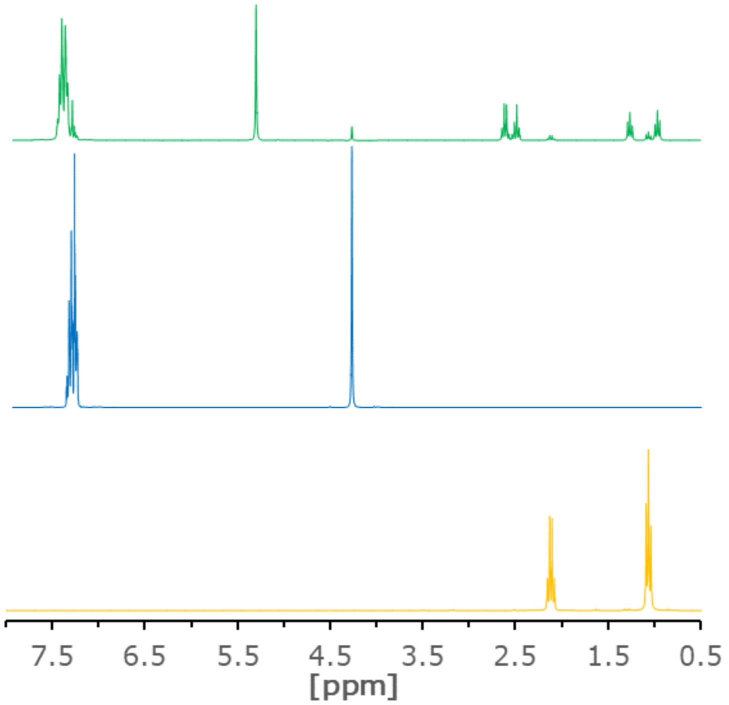

Figure S11. ^{1}H NMR data of [2+3] dipolar cycloaddition with **Cu-TAPB-BTCA** as the catalyst, 3-hexyne (yellow) as alkyne, and benzyl azide (blue). The reaction product (green).

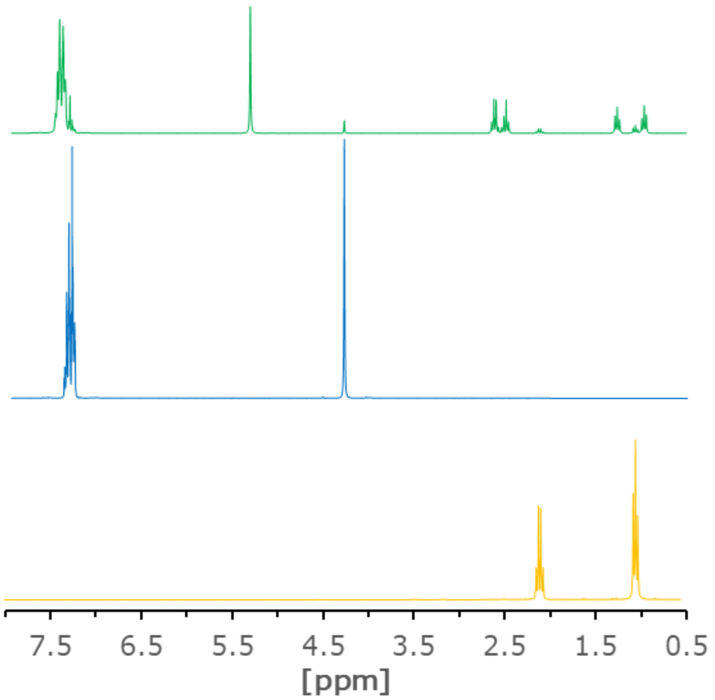

Figure S12. ^{1}H NMR data of [2+3] dipolar cycloaddition with **Cu-TAPB-TFP** as the catalyst, 3-hexyne (yellow) as alkyne, and benzyl azide (blue). The reaction product (green).

S7 Leaching Studies

58 µL (0.40 mmol) of benzyl azide was added to a suspension of 0.40 mmol of the alkyne and 5 mg of the corresponding COF in dichloromethane (1.5 mL) in a capped vial. The reaction mixture was stirred at 30 ºC for 9 h. Then the catalyst was removed by centrifugation, and the reaction was stirred for an additional 9 h at 30 ºC. Then the solvent was evaporated under vacuum, and the resulting oil was analysed by ^{1}H NMR.

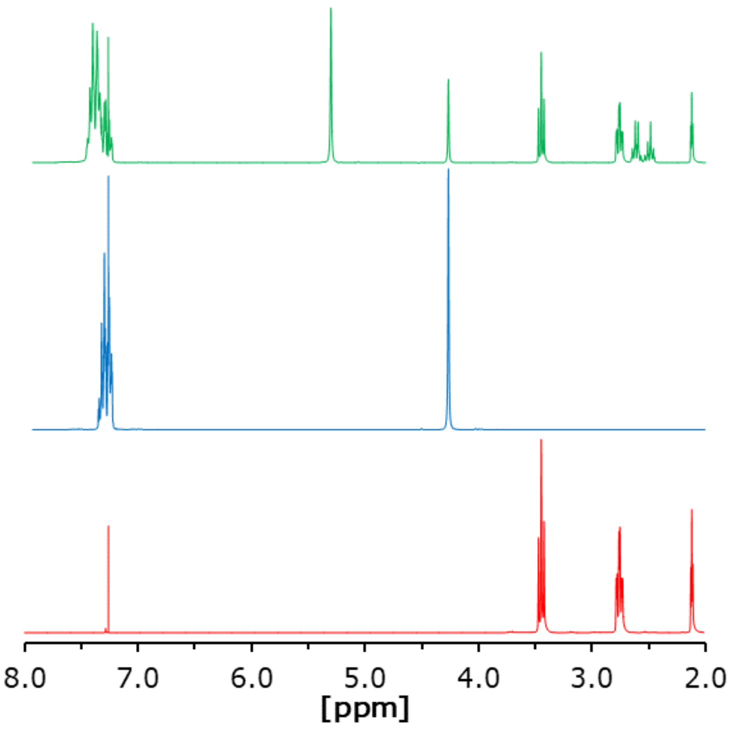

Figure S13. ^{1}H NMR data of leaching test with **Cu-TAPB-BTCA** as the catalyst, 4-bromobutyne (red) as alkyne, and benzyl azide (blue). The reaction product (green).

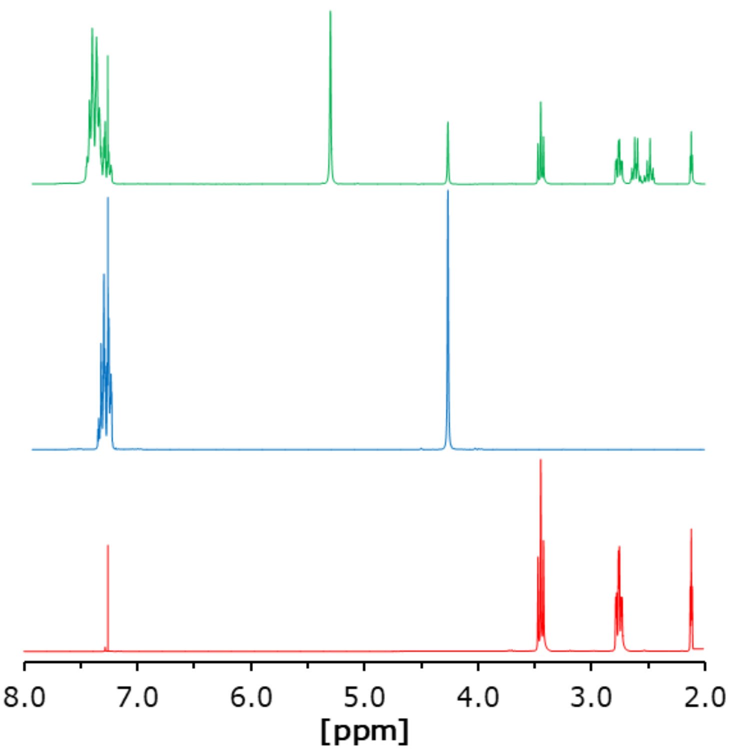

Figure S14. ^{1}H NMR data of leaching test with **Cu-TAPB-TFP** as the catalyst, 4-bromobutyne (red) as alkyne, and benzyl azide (blue). The reaction product (green).

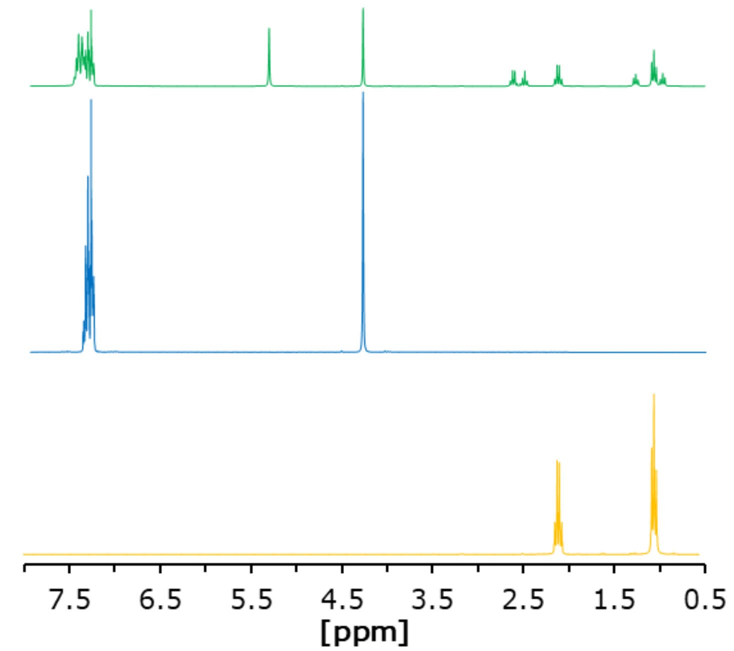

Figure S15. ^{1}H NMR data of leaching test with **Cu-TAPB-BTCA** as the catalyst, 3-hexyne (yellow) as alkyne, and benzyl azide (blue). The reaction product (green).

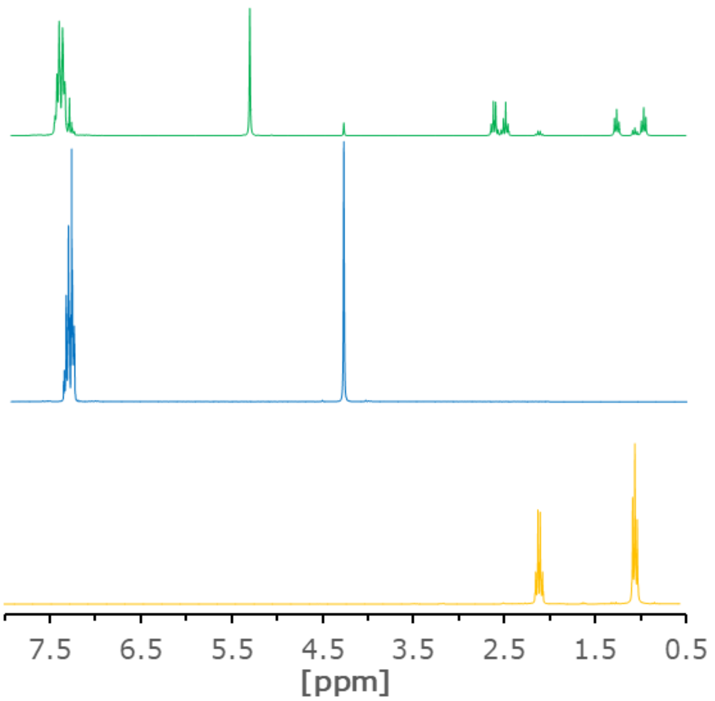

Figure S16. ^{1}H NMR data of leaching test with **Cu-TAPB-TFP** as the catalyst, 3-hexyne (yellow) as alkyne, and benzyl azide (blue). The reaction product (green).

S8 Postcatalytic Powder X Ray Diffraction and FE-SEM

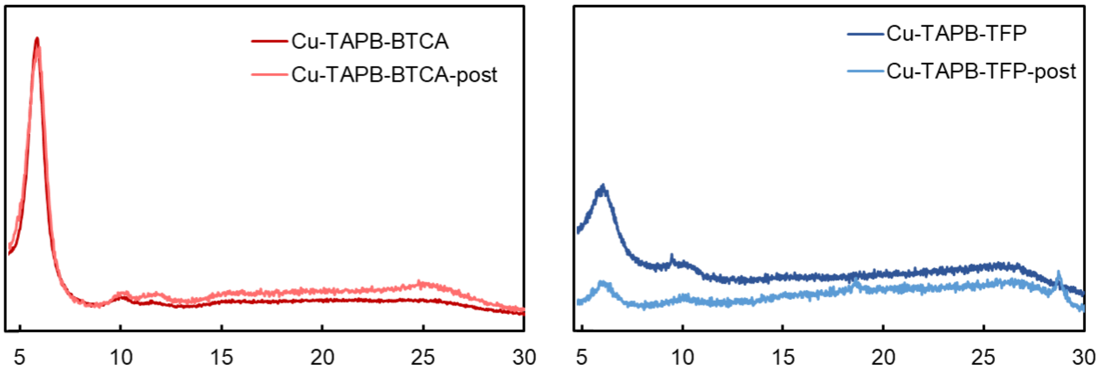

Figure S17. PXRD data of **Cu-TAPB-BTCA** and **Cu-TAPB-TFP** before catalysis and after the third run.

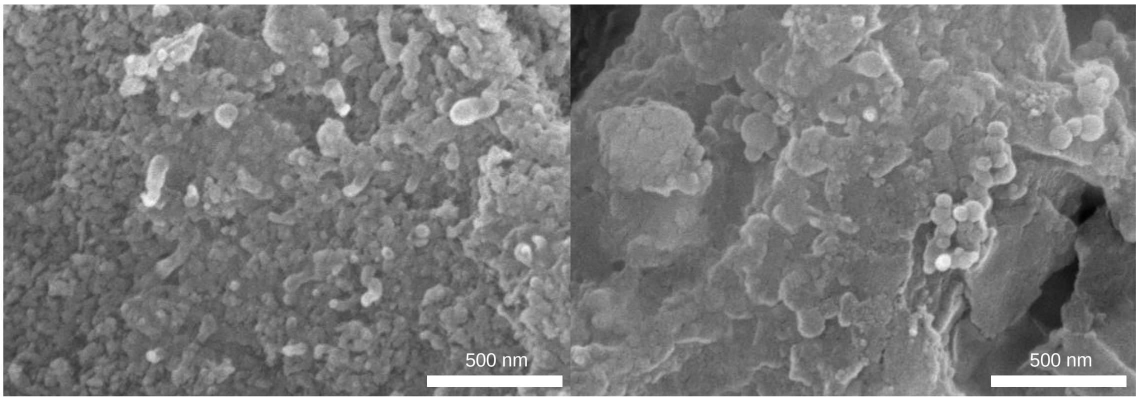

Figure S18. FE-SEM data of **Cu-TAPB-BTCA** before catalysis (left) and after the third run (right).

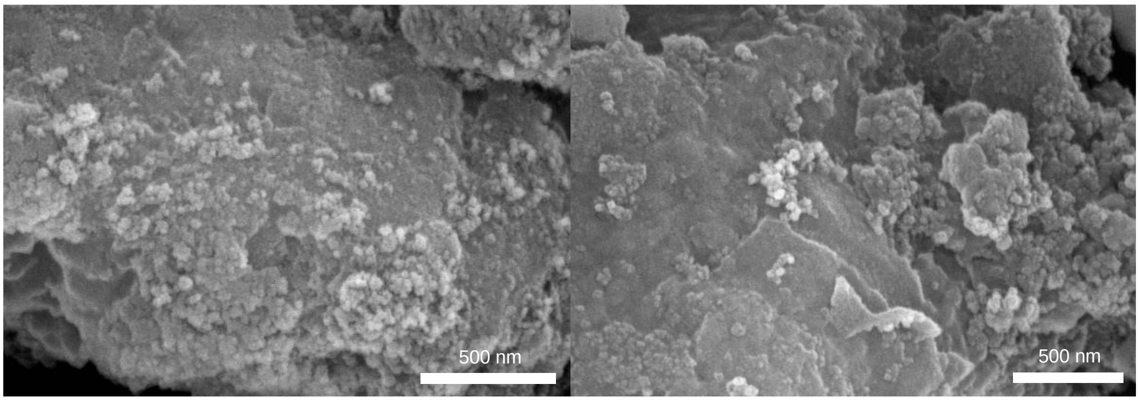

Figure S19. FE-SEM data of **Cu-TAPB-TFP** before catalysis (left) and after the third run (right).

References

[1] Romero-Muñiz, I.; Mavrandonakis, A.; Albacete, P.; Vega, A.; Briois, V.; Zamora, F.; Platero-Prats, A. E. Unveiling the Local Structure of Palladium Loaded into Imine-Linked Layered Covalent Organic Frameworks for Cross-Coupling Catalysis, *Angew. Chemie Int. Ed.* **2020**, 59, 13013-13020

[2] Fiandanese, V.; Iannone, F.; Marchese, G.; Punzi, A. A facile synthesis of N–C linked 1,2,3-triazole-oligomers *Tetrahedron* **2011**, *67*, 5254–5260.

[3] Candelon, N.; Lastécouères, D.; Diallo, A. K.; Ruiz Aranzaes, J.; Astruc, D.; Vincent, J. M. A highly active and reusable copper(i)-tren catalyst for the “click” 1,3-dipolar cycloaddition of azides and alkynes *Chem. Commun.* **2008**, *1*, 741–743.
